# Seroprevalence of *Rotavirus* infection in pig population of Arunachal Pradesh

**DOI:** 10.14202/vetworld.2016.1300-1304

**Published:** 2016-11-27

**Authors:** G. B. Garam, D. P. Bora, B. Borah, M. Bora, S. K. Das

**Affiliations:** Department of Microbiology, College of Veterinary Science, Assam Agricultural University, Khanapara Campus, Guwahati, Assam, India

**Keywords:** antibody, Arunachal Pradesh, pig population, *Rotavirus*, seropositive

## Abstract

**Aim:**

This study was conducted to find out the seroprevalence of *Rotavirus*(RV) infection among the pig population of Arunachal Pradesh.

**Materials and Methods:**

Serums samples were collected from piglets of age ranging from 1 week to 6 months and the sows associated with the piglets that were reared under organized and unorganized system of management in six different districts of Arunachal Pradesh. The prevalence of RV specific antibodies was detected using a polyclonal antibody-based indirect enzyme-linked immunosorbent assay (i-ELISA).

**Results:**

The study revealed that out of 394 serum samples, 255 (64.72%) samples were found to be positive for RV-specific antibody in i-ELISA. Considering the samples from different districts, Papumpare district of Arunachal Pradesh showed highest numbers of seropositive animals (68.75%) followed by upper Subansiri (64.91%) while West Siang district showed lowest positivity rate (61.22%).

**Conclusion:**

As considerable seropositivity was recorded among pig population of Arunachal Pradesh in this study, there is urgent need to establish high-impact and cost-effective public health intervention tools, key among them being the introduction of strict hygiene practice and RV vaccination program, to greatly reduce the number of deaths due to diarrheal diseases. To the authors’ knowledge, this is the first report on the prevalence of RV infection from pigs of Arunachal Pradesh.

## Introduction

*Rotavirus* (RV) infection is the leading cause of moderate to severe acute diarrheal disease in young animals [1]. Since its discovery in 1973, RV has been established as the most common cause of acute viral gastroenteritis among human infants and the neonates of most farm animal species worldwide [2,3]. In piglets, 25-30% mortality is seen during the preweaning period [4,5]. Among infectious agents associated with neonatal enteric disease, RV, *Coronavirus*, *Cryptosporidium*, and *Escherichia coli* collectively are responsible for 75-95% of infection in which RV alone accounts for about 27-36% [2]. RV belongs to the genus RV under the family Reoviridae[6]. They are nonenveloped, icosahedral particles consisting of eleven segments of double-stranded RNA enclosed in a triple-layered protein capsid [7]. RVs are classified into G-type and P-type based on the VP7 and VP4 structural genes, respectively [8]. RV is highly infectious and may be transmitted via the fecal-oral route and in respiratory droplets [9,10]. Infected viruses preferentially multiply in the intestinal epithelia and cause extensive damage to the enterocytes. This results in malabsorption leading severe to acute diarrhea [11].

Arunachal Pradesh, a North Eastern state of India, is a tribal state where there is no any taboo attached to the farming of pigs. Almost all rural household has minimum of one to two or more pigs in their backyard [12]. Pig meat (pork) is very popular among all the tribes of the state. Despite having enormous potential of pig farming in Arunachal Pradesh, due to lack of proper technical knowledge and guidance most of the pig farmers suffers heavy loss due to various kinds of diseases, of which neonatal diarrhea caused by RV is one of the most important diseases in piglets. The prevalence of RV infections in animals has been well documented from different parts of India [5,13,14]. However, no data on distribution of RV among pig population of Arunachal are available as no systematic study has been carried out so far. Studies conducted in Assam, a neighboring state of Arunachal Pradesh, have clearly indicated the presence of RV among pig population of the state [4,15]. In Assam, the overall prevalence of RV was found to be 41.5% where maximum numbers of positive cases were found in piglets (46.3%) followed by human (40%) and cattle (37.1%) [16]. To protect and reduce the prevalence of the disease, epidemiological studies in Arunachal Pradesh are of utmost importance besides developing technologies for the virus isolation, identification and above all molecular characterization of the virus for future vaccine strategy.

This study was conducted to determine the seroprevalence of RV infection in pig population of Arunachal Pradesh, with a view to have some baseline data to formulate control measures.

## Materials and Methods

### Ethical approval

Ethical approval for the study was obtained from IAEC, Assam Agricultural University (AAU), Khanapara campus vide approval No.770/ac/CPCSEA/FVSc/AAU/IAEC/14-15/263 dtd. 20.6.2014.

### Farms and animals

**Figure-1:**
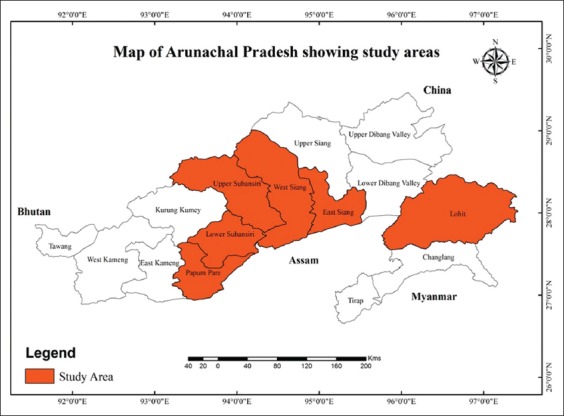
Map of Arunachal Pradesh showing the study areas. [Source: DIVA-GIS programme, URL: www.diva-gis.org].

The study was conducted in six districts of Arunachal Pradesh, *viz*., lower Subansiri, upper Subansiri, East Siang, West Siang, Papumpare, and Lohitwhere pig farming is commonly practiced and was accessible during the study period. The study area with the districts is depicted in Figure-1. The pig population in this area were both organized and unorganized farming. In organized farms, animals were maintained mostly on concrete floors while wooded floors are used in unorganized farms. Further, in organized farms, animals were reared following modern scientific managemental practices such as regular deworming, proper vaccination, etc. In unorganized farms, such practices were not followed. The piglets (2-4 months age) and corresponding sows (mothers’) were targeted for studying the RV prevalence. The serum samples were collected through the active participation of farmers and veterinarians working in the different location of Arunachal Pradesh both from organized and unorganized pig farms.

### Serum sample

Blood samples were collected from piglets having suspected RV-induced diarrheaand associated sows. The samples were obtained from the ear vein or cranial vena cava, and serum was separated by centrifugation (SIGMA, Model 3K30, UK). The samples were labeled properly, transported in ice-box to the laboratory and stored at −20°C for further use. A total of 394 numbers of serum samples were collected from the pig population of six districts of Arunachal Pradesh.

### Detection of anti-RV antibody in serum

Anti-RV antibodies in collected serum samples were detected using an indirect-enzyme-linked immunosorbent assay (i-ELISA) as per method described byHohdatsu *et al*. [17].

### Viral antigen

Standard Group A RV maintained in the Department of Microbiology, College of Veterinary Science, AAU, Khanapara, Guwahati was used as coating antigen in the i-ELISA.

### i-ELISA

Antibodies to RV in the serum sample were detected and titrated by i-ELISA as per the method of Hohdatsu *et al*. [18]. Revalidation of the test was done using standard RV antigen and pig anti-RV antibody. The standard RV antigen was used as the coating antigen. Freeze-dried virus was reconstituted in 0.5 ml of distilled water. The working virus dilution was determined following chequerboard titration technique, and the virus titer 1:100 was used as coating antigen in the test.

The test was performed in flat bottom, 96 well microtiter plates (Nunc). The wells were coated with 50µl of 1:100 dilution of RV antigen in carbonated-bicarbonate buffer (pH 9.6) and incubated overnight at 4°C. The plates were washed 4-5 times with Tween-phosphate buffered saline (PBS-T) and two-fold serial dilution of the serum sample was done with initial dilution of 1:10 in blocking buffer containing 5% lactalbuminhydrolysate in 0.5 M PBS-T. Plates were incubated at 37°C for 1 h. The plate was washed again as before. Rabbit anti-swine HRPO conjugate (1:1000, DAKO, Denmark) was added in 50 µl volume to all the wells and incubated at 37°C for 1 h. Washing was repeated as before. To visualize the amounts of bound conjugate, 50 µl of freshly prepared substrate (H_2_O_2_) and chromogen (OPD, Sigma) mixture was added to the wells and allowed to react for 15 min. Reaction was stopped by adding 50 µl of 1M H_2_SO_4_ to the wells. The cut-off OD value (OD of positive − OD of negative control) ≥0.1 was considered as positive, and the highest dilution of serum showing positive result was taken as antibody titer of the serum sample. Serum of colostrum deprived piglet was used as negative control in the test.

## Results

**Figure-2:**
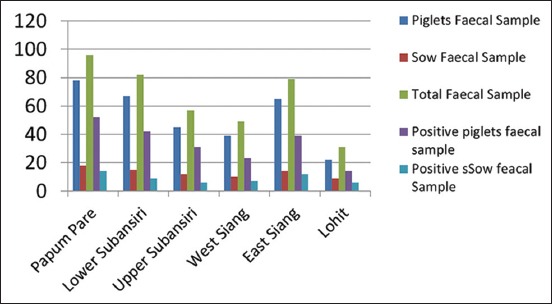
Percent prevalence of rotavirus-specific antibodies in different districts of Arunachal Pradesh.

Serum samples collected both from piglets (n=318) and sows (n=78) were tested for the presence of anti-RV specific antibody by i-ELISA (Table-1). Out of 394 serum samples, 255 (64.72%) were found positive for RV antibodies. Considering the age groups, the highest prevalence of RV-specific antibodies (69.23%) was found in adult animals (sows). Considering the samples from different districts, Papumpare district of Arunachal Pradesh showed highest numbers of seropositive animals (68.75%) followed by upper Subansiri (64.91%) while West Siang district showed lowest positivity rate (61.22%). Details of the result are presented graphically in Figure-2.

**Table 1:** Prevalence of RV-specific antibodies in the serum sample of piglets and sows.

Districts	Number of serum sample		Total	Number of positive		Positive (%)
	
	Piglets	Sows		Piglets	Sow	
Papum Pare	78	18	96	52	14	66 (68.75)
Lower Subansiri	67	15	82	42	9	51 (62.19)
Upper Subansiri	45	12	57	31	6	37 (64.91)
West Siang	39	10	49	23	7	30 (61.22)
East Siang	65	14	79	39	12	51 (64.55)
Lohit	22	9	31	14	6	20 (64.51)
Overall	318	78	394	201	54	255/394 (64.72)

RV=*Rotavirus*

## Discussion

RV infection is known to be the major cause of enteritis and diarrhea in neonates of most animal species including human infants [14]. However, infection with RV in animal population in a particular area may vary depending on the management practices and the type of farming [1,2]. Serological surveys carried out in various countries showed that 70-100% adult pigs were seropositive for swine Group A, B and C RV [18,19]. Arunachal Pradesh, a North Eastern state of India, has substantial population of pigs throughout the districts under study [20]. However, there is no data available on the prevalence of RV infection from pig population of Arunachal Pradesh. Results of the present serological study carried out in pigs of Arunachal Pradesh have shown that RV specific antibodies were prevalent in 64.72% of pigs. Higher prevalence of RV and transmissible gastroenteritis virus antibodies in intensive pig breeding farms was reported by various workers [21,22]. Further reports of RV prevalence among pig population of Assam, a neighboring state of Arunachal Pradesh are available [23].

Considering the age groups, the highest prevalence of RV-specific antibodies (69.23%) was found in adult animals (sows). This is in agreement with the findings of earlier study carried out by Nath[22]. Higher prevalence of RV antibodies in adult pigs might be due to repeated exposure to RV infection at different stages of their life. Highest seroconversion rate (up to 1:640) recorded in adult pigs in the present study also supported this view. In piglets also, a highseropositivity (63.20%) for RV was detected in the present study. In preweaned pigs, RV specific antibodies were recorded up to 72.5% and piglets also possessed comparatively higher antibody titer (up to 1:160). High percentage of seropositive clearly indicated that piglets acquire RV specific antibody via colostrum. These findings are in accordance with that of Hess and Bachmann [24]. They also reported a high percentage of RV seropositivity in piglets, which was due to colostralantibody. Considering the samples from different districts, Papumpare district of Arunachal Pradesh showed highest numbers of seropositive animals (68.75%) followed by upper Subansiri (64.91%) while West Siang district showed lowest positivity rate (61.22%). Higher seropositivity in the said districts may be due to the more pig population density in these districts in comparison to the other districts of Arunachal Pradesh [20] and the unorganized type of farming followed by the farmers as reported by earlier worker [22].

## Conclusion

This study confirms the prevalence of RV infection among the pig population of Arunachal Pradesh. However, further studies are needed to elucidate the transmission, epidemiology, and characterization of the prevalent RV in the pig population ofArunachal Pradesh. Furthermore, there is urgent need to establish high-impact and cost-effective public health intervention tools, key among them being the introduction of strict hygiene practice and RV vaccination program, to greatly reduce the number of deaths due to diarrheal diseases.

## Authors’ Contributions

This study was a part of GBG’s research work during his M.V.Sc. program. GBG carried out the experiment. DPB and SKD designed the experiment and provided necessary guidelines. MB and BB assisted in laboratory works. DPB drafted the final manuscript. All authors have read and approved the final manuscript.
